# Increased Serum Oxidative Stress Markers in Women with Uterine Leiomyoma

**DOI:** 10.1371/journal.pone.0072069

**Published:** 2013-08-09

**Authors:** Pietro Santulli, Bruno Borghese, Herve Lemaréchal, Mahaut Leconte, Anne-Elodie Millischer, Frédéric Batteux, Charles Chapron, Didier Borderie

**Affiliations:** 1 Université Paris Descartes, Sorbonne Paris Cité,Faculté de Médecine, AP- HP, Hôpital Cochin, Department of GynecologyObstetrics II and Reproductive Medicine,75679 Paris, France; 2 Université Paris Descartes, Sorbonne Paris Cité,Faculté de Médecine, AP-HP, Hôpital Cochin, Laboratoired’immunologie, EA 1833, 75679 Paris, France; 3 Université Paris Descartes, Sorbonne Paris Cité,Faculté de Médecine, INSERM, Unité de recherche U1016, Institut Cochin, CNRS (UMR 8104), Paris, France; 4 Université Paris Descartes, Sorbonne Paris Cité,Faculté de Médecine, AP-HP, Hôpital Cochin, Laboratory of Biochemistry, Paris, France; Queensland Institute of Medical Research, Australia

## Abstract

**Background:**

Uterine leiomyomas (fibroids) are the most common gynaecological benign tumors in premenopausal women. Evidences support the role of oxidative stress in the development of uterine leiomyoma. We have analysed oxidative stress markers (thiols, advanced oxidized protein products (AOPP), protein carbonyls and nitrates/nitrites) in preoperative sera from women with histologically proven uterine leiomyoma.

**Methodology/Principal Findings:**

We conducted a laboratory study in a tertiary-care university hospital. Fifty-nine women with histologically proven uterine leiomyoma and ninety-two leiomyoma-free control women have been enrolled in this study. Complete surgical exploration of the abdominopelvic cavity was performed in each patient. Preoperative serum samples were obtained from all study participants to assay serum thiols, AOPP, protein carbonyls and nitrates/nitrites.

Concentrations of serum protein carbonyl groups and AOPP were higher in leiomyoma patients than in the control group (p=0.005 and p<0.001, respectively). By contrast, serum thiol levels were lower in leiomyoma patients (p<0.001). We found positive correlations between serum AOPP concentrations and total fibroids weight (r=0.339; p=0.028), serum AOPP and serum protein carbonyls with duration of infertility (r=0.762; p=0.006 and r=0.683; p=0.021, respectively).

**Conclusions/Significance:**

This study, for the first time, reveals a significant increase of protein oxidative stress status and reduced antioxidant capacity in sera from women with uterine leiomyoma.

## Introduction

Uterine leiomyomas, also called uterine fibroids or myomas, are the most common benign gynecological tumors of women in reproductive age [1].

The most common symptoms of uterine leiomyomas are heavy uterine bleeding and pelvic pain [2]. They are associated with infertility and adverse birth outcomes including recurrent miscarriage and fetal death [2]. They cause significant morbidity, and are the most commonly cited reason for hysterectomy [3–5]. Leiomyomas are considered a major health related problem, responsible for total direct and indirect annual costs of $5.89-$34.37 billions in the United States [6]. Surprisingly, given the high prevalence and serious impact on women’s health, very little is known about the pathogenesis of this disease [7].

Uterine leiomyomas are benign tumors arising from the smooth muscle cells of the myometrium. They contain large amounts of extracellular matrix (collagen, proteoglycan, fibronectin) and are surrounded by a thin pseudocapsule of areolar tissue, containing feeding vessels, and compressed muscle fibers [3].

Two essential features of leiomyomas are an increase in smooth muscle cell proliferation and an excessive deposition of extracellular matrix [8]. The fibroid pathogenesis is multifactorial, and the precise mechanisms involved in its initiation and growth remain unclear. Several factors might be involved in the enlargement of leiomyomas, including interactions between various genes, hormones, growth factors and cytokines [9] although estrogen and progesterone have been traditionally considered as the majors promoters of leiomyoma growth [10,11].

For example, an inadequate antioxidant protection or an excessive production of reactive oxygen species (ROS) can alter the cellular oxidative balance creating a condition knows as oxidative stress [12].

Oxidative stress plays a key role in numerous diseases such as chronic inflammatory diseases [12,13]. In endometriosis, a metastatic benign disease [14], endometriotic cells display an increase in endogenous oxidative stress with ROS produced in excess and failures in ROS detoxification pathways [15,16]. In neoplastic conditions, the intracellular redox status controls tumor cell proliferation [17,18], and enhances the metastatic potential of tumor cells [19].

Estrogen as well as estrogen metabolites have been reported to act as pro-oxidants [20]. Estrogen-induced ROS play important roles in cell transformation, cell proliferation, migration and invasion by increasing genomic instability and influencing redox sensitive transcription factors [21]. Although several reports have suggested the role of the oxidative stress in the development of uterine leiomyomas [22–25], no study has investigated the markers of oxidative stress in the sera of patients. Such study could be useful to establish a relationship between the development of the tumor and to determine the pathways involved.

In the present study we assayed serum oxidative stress markers in a large series of women with uterine leiomyomas. The concentrations of these markers in patients were compared to those in women with no leiomyoma. The results were evaluated with respect to the number, size and weight of the uterine leiomyomas.

## Methods

### Patients

The ethics committee of our institution (CPP: Comité de Protection des Personnes) approved the study protocol. A cross-sectional study was conducted from January 2005 to December 2010, including a continuous series of 151 patients after providing informed written consent. A thorough surgical examination of the abdominopelvic cavity was performed in all study participants.

All patients underwent transvaginal ultrasonography (TVUS) during the month preceding the surgery. TVUS was performed with a Toshiba ultrasound machine, using a 5–9 MHz transducer [26]. All scans were performed without bowel preparation by a single radiologist (AEM) with more than 10 years of extensive gynecological experience. The radiologist was blinded to the results of clinical findings and previous imaging examinations. Each examination was interpreted in real time. The precise number and dimensions of fibroids were recorded at this time. Measurement of each fibroid was performed in three perpendicular diameters (sagittal, coronal and axial) [27]. For each myoma, the largest dimension was retained for the study [28]. Women with symptomatic uterine leiomyomas at the TVUS examination were allocated to the leiomyoma group (study group) and were surgically treated by myomectomy (n=59). All women in the study group had histologically proven uterine leiomyoma. For each patient, the following parameters were recorded: total number of leiomyomas, total weight (g) and total size (cm. The total weight corresponds to the weight of each detectable uterine fibroid removed and measured before histopathological analysis. The total size corresponded to the sum of the largest diameters (in cm) of each fibroid.

Women without any evidence of uterine leiomyoma as checked during preoperative TVUS [28] were allocated to the control group (n=92). In this group, the indications for surgery were non-endometriotic benign ovarian cysts and tubal infertility.

All women were < 42 years old. Ovarian malignancy and borderline tumours were not included in this study. No woman in this study had a previous history of myomectomy, autoimmune or inflammatory disease. Women with virus C or virus B hepatitis or human immunodeficiency virus infection were not included in this study. None of included women was pregnant.

Because endometriotic cells display an increased endogenous oxidative stress and alterations in ROS detoxification pathways [15,16], women with macroscopic endometriosis during abdomino-pelvic surgical exploration, past history of hormonal and/or surgical treatment for endometriosis, were excluded of the study.

The study analysis used a prospectively managed database [29]. For each patient, personal history data were obtained during face-to-face interviews conducted by the surgeon during the month preceding surgery. We used a highly structured questionnaire previously published [30]. The following data were recorded: age, parity, gravidity, height, weight, previous history of infertility, existence of gynecologic pain [dysmenorrhea, deep dyspareunia, non cyclic chronic pelvic pain (NCCPP)] [31], gastrointestinal (GI) [32] and lower urinary (LU) tract symptoms [33]. Pain intensity was evaluated preoperatively using a 10-cm visual analogue scale (VAS) [34]. Infertility was defined as at least 12 months of unprotected intercourse not resulting in pregnancy [35]. The duration of menstrual bleeding, recorded in each patient, corresponded to the number of days requiring the use of tampons or pads. Biological features of inflammation such as C-reactive protein (mg/l), white blood cell count (U/ml) were also collected for each patient.

### Collection of serum

Serum samples were collected from all participants during the month that preceded surgery. Briefly, samples of 5–10 ml of venous blood were collected through a peripheral venous catheter (PVC) then centrifuged at 800 g for 12 min at 4 °C. Serum supernatants were collected, aliquoted, and stored at -70 °C until use.

### Measurement of stress oxidative parameters

#### Thiols

Thiols were determined with Ellman’s reagent [36]. Fifty µL of plasma were mixed with 1.0 mL of 0.1 M Tris, and 10 mM EDTA at pH 8.2. The absorbance was determined at 412 nm, and 40 µL of 10 MmEllman’s reagent (Sigma) in methanol were added to the sample. The absorbance obtained before the addition of Ellman’s reagent was subtracted from that obtained after incubation with Ellman’s reagent. A control containing Ellman’s reagent only was included, and the concentration of thiol groups was calculated using a molar extinction coefficient of 13,600 M^-1^ cm^-1^ at 412 nm.

#### Advanced oxidation protein products (AOPP)

AOPP were quantified as described previously [37]. We placed 200 µl of serum diluted 1:5 in phosphate-buffered saline into each well of a 96-well microtitre plate and added 20 µl of acetic acid to each well. For the standard, we added 10 µl of 1.16 M potassium iodide (Sigma, St Louis, MO, USA) to 200 µl of chloramine-T solution (0 to 100 µmol/l) (Sigma, St Louis, MO, USA) in a well and then 20 µl of acetic acid. The absorbance of the reaction mixture was immediately read at 340 nm against a blank consisting of 200 µl of phosphate-buffered saline, 10 µl of 1.16 M potassium iodide, and 20 µl of acetic acid. AOPP concentrations are expressed as micromoles/liter of chloramine-T equivalents.

#### Carbonyl groups

Protein carbonyl groups were detected and quantified using 2,4-dinitrophenylhydrazine (DNPH) [38]. Briefly, 0.5 ml serum (1 mg protein/ml) were treated with 0.5 ml 10 mM DNPH in 2 M HCl, or with 0.5 ml 2 M HCl alone for the blank. Samples were incubated for 1 h at room temperature in the dark, and then treated with 10% trichloroacetic acid and centrifuged. The pellet was washed three times in ethanol/ethyl acetate and solubilized in 1 ml of 6 M guanidine in 20 mM potassium phosphate, adjusted to pH 2.3 with trifluoracetic acid; the resulting solution was incubated at 37° C for 15 min. The carbonyl concentration was determined from the difference in absorbance at 370 nm between DNPH-treated and HCl-treated samples, with ε_370_ =22,000 M^-1^ cm^-1^. The carbonyl content was expressed as nanomoles of carbonyl per milligram of protein.

#### Nitrates plus nitrites

The nitrate concentration was determined with a spectrophotometric assay using oxidation catalyzed by cadmium metal which converts nitrates to nitrites as previously described [39]. The total nitrites thus determined correspond to NO production. Proteins were first removed from the medium by incubation with zinc sulphate. Cadmium metal was then added and the medium incubated overnight with shaking in the dark. The next day, the solution was mixed and sulphanilamide and N-1 naphtylethylenediamine were added. These compounds form a colored complex with nitrites, which can be assayed by spectrophotometry (Dynatech Instruments, Guernesey). The detection limit was 0.04 µmol/l.

### Statistical analysis

All data were collected in a computerized database and analyzed by SPSS software (SPSS Inc., Chicago, IL). Student’s t-test was used for quantitative variables and Pearson’s chi-square or Fisher’s exact test for qualitative variables as appropriate.

Considering the non gaussian distribution of serum stress oxidative parameters, the statistical analysis between two groups was performed with the Mann–Whitney U Test. Correlations between serum stress oxidative parameters and semiquantitative clinical, biological and anatomical data were determined using the nonparametric Spearman’s rank correlation test. P<0.05 was considered statistically significant.

## Results

### Patients and controls

Fifty nine women with leiomyomas and 92 leiomyoma-free women were recruited for this study (Flowchart S1). The major clinical, laboratory and surgical features of the two groups are presented in Table 1. For all participants in the study (n=151), a thorough surgical examination of the abdominopelvic cavity was performed. Among the 59 histologically proven leiomyoma patients, the mean number of leiomyoma was 6.5 ± 6.5 for each women. The mean total weight of uterine leiomyoma was 292.8 ± 275.7 g, and the mean total size was 17.9 ± 11.4 cm. Among the 92 leiomyoma-free women, intra-operative and histopathological findings are resumed as follows: 51 (55.4%) benign ovarian cysts (30 dermoid, 7 serous, 8 mucinous cysts), 17 (18.5%) paratubal cysts and 24 (26.1%) tubal defects (11 pelvic tubo-ovarian adhesion, 5 hydrosalpinxs and 8 proximal tubal blockage). There were no differences in age, gravidity and parity between the study and control groups (Table 1). Women in the leiomyoma group displayed a longer duration of menstrual bleeding as compared to disease-free women (Table 1). The percentages of patients with hormonal treatment were similar in both groups. Biological features of inflammation, such as mean C-reactive protein and mean white blood cell counts were within reference ranges for both groups without statistical difference between the study and the control groups (Table 1).

**Table 1:** Baseline characteristics.

	**Fibroids (N=59)**	**Controls (N=92)**	**P**
**Patient characteristics**			
Age (years)	33.2 ± 3.8	32.7 ± 5.6	0.508
Height (cm)^a^	164.8 ± 6.8	165.6 ± 6.1	0.428
Weight (kg)^a^	66.8 ± 12.0	63.1 ± 11.4	0.060
Parity^a^	0.3 ± 0.6	0.4 ± 0.8	0.417
Gravidity^a^	1.0 ± 1.1	0.8 ± 1.3	0.283
Duration of menstrual bleeding (days)^a^	6.0 ± 2.8	4.9 ± 1.5	<0.001
**Preoperative painful symptoms scores: ^a^^b^^c^**			
Dysmenorrhea	4.7 ± 3.3	3.7 ± 3.1	0.068
Dyspareunia^$^	2.5 ± 3.0	1.8 ± 3.0	0.177
Non cyclic chronic pelvic pain	1.0 ± 2.1	1.6 ± 2.7	0.138
Gastrointestinal symptoms	1.1 ± 2.4	0.5 ± 1.7	0.103
Lower urinary symptoms	0.1 ± 0.7	0.1 ± 0.7	0.932
**Laboratory findings :**			
CRP (mg/l)	1.4 ± 0.8	2.1 ± 3.2	0.122
WBC (U/mL)	5800,0 ± 2239.5	6516.7 ± 1732.8	0.103
**Fibroids characteristics:**			
Total weight (g)	292.8 ± 275.7	NA	NA
Total size (cm)	17.9 ± 11.4	NA	NA
Total number	6.5 ± 6.5	NA	NA

^a^Data are presented as mean ± standard deviation;

^b^ Sometimes more than one for the same patient;

^c^ Visual analogue scale;

^t^ Student’s t-test;

^k^ Pearson’s chi-square test;

^$^ 4% of patients have no sexual intercourse at the moment of the surgery.

NA: not applicableWBC count: white blood cell count

WBC count: white blood cell count

### Serum oxidative stress markers

**Figure 1:**
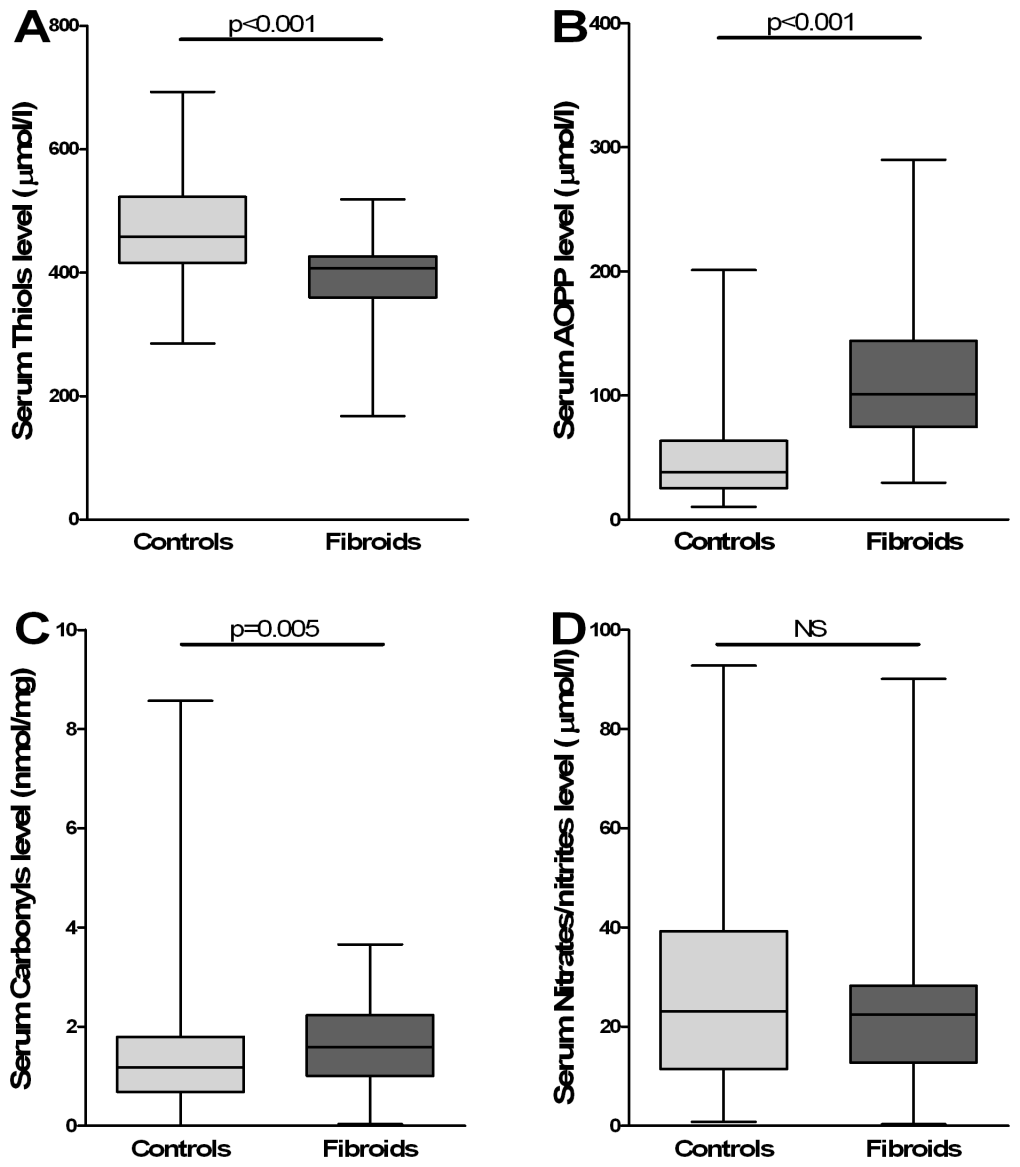
Serum oxidative stress parameters in women with uterine leiomyoma and in controls. **A**) Serum Thiols (µmol/l). **B**) Serum AOPP (µmol/l). **C**) Serum protein carbonyls (nmol/mg). **D**) Serum Nitrates/Nitrites (µmol/l). Statistical analysis was performed using the Mann-Whitney U. Serum oxidative stress parameters are represented as boxes and whisker plots. The bottom and top of the boxes represent the interquartile range (the 25th and 75th percentile, respectively), and the band near the middle of the box is the median. The ends of the whiskers represent the minimum and maximum of all the data. P < 0.05 was considered statistically significant.

Serum Thiols, AOPP, protein carbonyls and nitrates/nitrites were measured in the sera of all the 151 women studied. Concentrations of serum protein carbonyl groups and AOPP were higher in leiomyoma patients than in the control group (p=0.005 and p<0.001, respectively) whereas serum thiols levels were significantly lower (p<0.001) (Table 2). Figure 1 depicts the median of serum protein carbonyls, AOPP and thiols concentrations in leiomyoma and controls. Concentrations of serum nitrates/nitrites were not different between leiomyoma and control groups (Table 2 and Figure 1).

**Table 2:** Statistical analysis for serum oxidative stress parameters in women with uterine fibroids and controls.

	**Fibroids (n=59)**	**Controls (n=92)**	**P**
**Thiols (µmol/l)**	407.2 (168.3-519.1)	458.4 (285.7-693.2)	<0.001^u^
**AOPP (µmol/l)**	101.1 (29.8-290.0)	38.4 (10.3-201.2)	<0.001^u^
**Carbonyls** (**nmol/mg**)	1.6 (0.0-3.7)	1.2 (0.0-8.6)	0.005^u^
**Nitrates / nitrites (µmol/l)**	22.4 (0.3-90.1)	23.1 (0.8-92.7)	0.703^u^

Results Are Expressed as Median (Range)

^u^ Statistical analysis was performed with the Mann–Whitney U test.

AOPP : advanced oxidation protein products

Even if the percentage of women with hormonal treatment was similar in both leiomyoma and control groups [10/59 (16.9%) *versus* 21/92 (22.8%), p=0.383], we studied the effect of hormonal treatment on the serum oxidative stress markers. Subgroup analysis showed no difference between patients with leiomyomas and hormonal treatment and those with no hormonal treatment in terms of serum thiols, AOPP, protein carbonyls and nitrates/nitrites (p=0.746; p=0.407; p=0.558; p=0.592, respectively). In the control group, the subgroup analysis showed no effect of hormonal treatment on serum thiols, AOPP, protein carbonyls and nitrates/nitrites (p=0.283; p=0.387; p=0.350; p=0.900, respectively). Among cycling women, 18.3% (n=22) did not provide information about their menstrual cycle phase. The percentages of cycling women with leiomyomas and of cycling controls in the proliferative phase were similar (48.8% (n=20) and 49.1% (n=28), respectively, p=0.655). The percentages of cycling women with leiomyomas and of cycling controls in the secretory phase were also similar (51.2% (n=21) and 50.9% (n=29), respectively, p=0.973). We studied the effects of menstrual cycle phases on serum protein oxidative stress markers in both study groups. We assessed hormonal treatment and menstrual cycle phases as an effect modifier, performing subgroup analysis in leiomyoma and control group (Table 3). As compared to controls women, Thiols serum levels were significantly lower in leiomyoma women using hormonal treatment or during the secretory phase (p=0.004 and p<0.001, respectively). By contrast serum AOPP levels were significantly increased in leiomyoma women with hormonal treatment and during both proliferative and secretory phases (p=0.001, p<0.001 and p<0.001, respectively). In addition serum carbonyl levels were significantly increased in leiomyoma woman during the proliferative phase as compared to controls women (p=0.045).

**Table 3:** Serum oxidative stress parameters in women with uterine fibroids and controls, according to hormonal therapy and menstrual cycle phases.

	**Phases**	**Fibroids**	**Controls**	**P**
	*Hormonal treatment*	401.5 (228.7–470.7) n=10	491.2 (303.8–569.4) n=21	0.004^m^
**Thiols (µmol/l)**	*Proliferative phase*	411.4 (311.6–519.1) n=20	428.8 (350.6–645.7) n=28	0.122^m^
	*Secretory phase*	402.1 (282.1–485.0) n=21	496.2 (285.7–693.2) n=29	<0.001^m^
	*Unknown*	408.0 (168.3–484.1) n=8	442.7 (382.2–548.8) n=14	0.056^m^
	*Hormonal treatment*	98.1 (38.1–128.5) n=10	30.0 (18.8–201.2) n=21	0.001^m^
**AOPP (µmol/l)**	*Proliferative phase*	116.2 (33.9–290.0) n=20	33.6 (10.3–107.2) n=28	<0.001^m^
	*Secretory phase*	98.1 (29.8–250.0) n=21	45.4 (14.5–158.5) n=29	<0.001^m^
	*Unknown*	103.6 (66.4–187.5) n=8	48.0 (18.2–132.2) n=14	0.008^m^
	*Hormonal treatment*	1.5 (0.4–2.5) n=10	1.1 (0.1–2.5) n=21	0.163^m^
**Carbonyls (nmol/mg)**	*Proliferative phase*	1.9 (0.3–3.0) n=20	1.3 (0.4–8.6) n=28	0.045^m^
	*Secretory phase*	1.4 (0.0–3.7) n=21	1.5 (0.0–2.8) n=29	0.914^m^
	*Unknown*	1.8 (0.8–2.9) n=8	0.9 (0.3–2.0) n=14	0.008^m^
	*Hormonal treatment*	25.5 (1.4–76.6) n=10	24.4 (1.6–70.8) n=21	0.933^m^
**Nitrates / nitrites (µmol/l)**	*Proliferative phase*	22.4 (1.2–90.1) n=20	23.1 (0.8–92.7) n=28	0.615^m^
	*Secretory phase*	22.4 (0.3–82.4) n=21	21.2 (1.7–70.6) n=29	0.860^m^
	*Unknown*	12.8 (7.0–28.2) n=8	18.7 (3.7–64.4) n=14	0.152^m^

Eight (13.6%) and fourteen (15.2%) women in the leiomyoma group and control group, respectively, did not provide information about their cycle menstrual phase or hormonal treatment (Unknown group)

Data: median (range)

^m^ Statistical analyses were performed with the Mann-Whitney test

AOPP : advanced oxidation protein products

According to the inclusion criteria, the women assigned to the control group were more likely to have a past history of infertility. Indeed, 18.6% (n=11) women with uterine leiomyoma told a previous history of infertility, *versus* 39.1% (n=36) women in the control group (p=0.007). However the duration of infertility was not different between the leiomyoma and the control groups (39.6 ± 34.9 months *versus* 45.6 ± 32.7 months; p=0.617). Among women with leiomyomas, serum thiols, AOPP, protein carbonyls and nitrates/nitrites concentrations were similar whether the patients had a past history of infertility or not (p=0.284; p=0.838; p=0.134; p=0.675, respectively). The same was observed in the control group (p=0.064; p=0.551; p=0.436; p=0.094, respectively). In addition we assessed past history of infertility, as an effect modifier, performing subgroup analysis in leiomyoma and control group according to the existence or not of previous history of infertility (Table S1).

### Correlations between serum oxidative stress markers and clinical and anatomical characteristics of leiomyomas

Correlations between serum oxidative stress markers and clinical and anatomical characteristics of leiomyomas are reported in Table 4.

**Table 4:** Correlation analysis of serum oxidative stress parameters and clinical data in women with fibroids.

	Spearman Rank Correlation Coefficient (r) (p value)			
Measurements	**Thiols**	**AOPP**	**Carbonyls**	**Nitrates/Nitrites**
Age	-0.026 (0.843)	0.106 (0.425)	0.106 (0.425)	-0.045 (0.737)
Height	-0.113 (0.394)	-0.052 (0.694)	-0.060 (0.652)	0.128 (0.333)
Weight	-0.185 (0.162)	0.075 (0.571)	-0.172 (0.192)	0.053 (0.690)
Infertility duration	-0.415 (0.233)	0.762 (0.006)	0.683 (0.021)	0.598 (0.068)
Duration of menstrual bleeding	-0.147 (0.276)	0.118 (0.382)	-0.034 (0.802)	0.078 (0.563)
Dysmenorrhea	-0.003 (0.982)	0.051 (0.702)	-0.134 (0.317)	-0.108 (0.420)
Deep dyspareunia	0.110 (0.427)	-0.047 (0.737)	0.055 (0.690)	-0.114 (0.413)
Non cyclic chronic pelvic pain	0.042 (0.752)	-0.034 (0.798)	0.018 (0.892)	0.082 (0.541)
Gastrointestinal symptoms	-0.123 (0.356)	0.144 (0.282)	0.010 (0.940)	-0.086 (0.522)
Lower urinary tract symptoms	0.107 (0.425)	0.028 (0.837)	-0.004 (0.976)	0.036 (0.790)
Total Fibroids Weight	-0.180 (0.254)	0.339 (0.028)	0.190 (0.227)	0.121 (0.443)
Total Fibroids Size	0.052 (0.764)	0.059 (0.733)	0.057 (0.741)	0.109 (0.525)
Total Fibroids Number	0.025 (0.854)	0.114 (0.406)	0.156 (0.255)	0.046 (0.738)

No correlations were found between pain symptom scores and serum oxidative stress parameters, independently of the type of pelvic pain symptoms.

**Figure 2:**
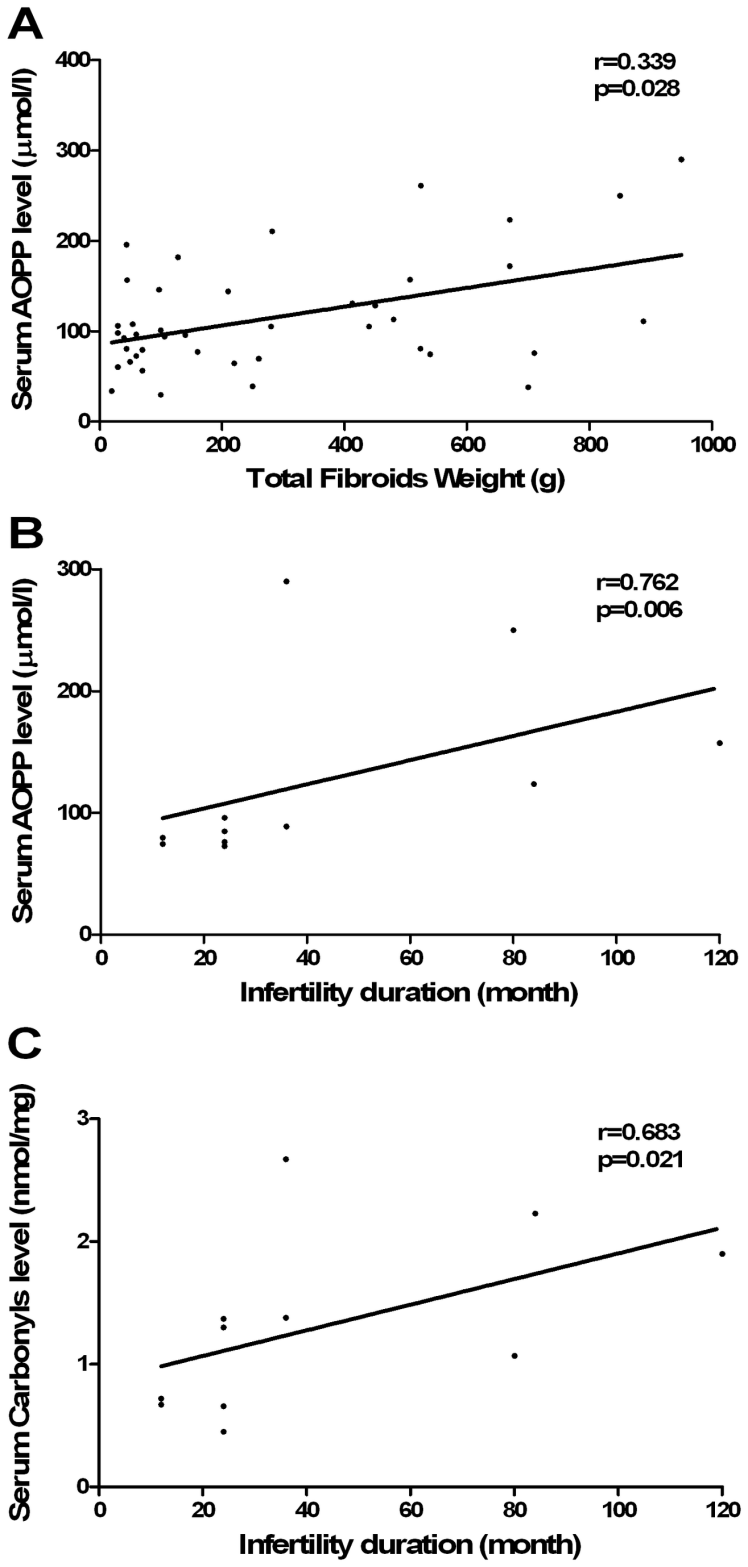
Correlation between serum oxidative stress parameters and anatomical parameters of uterine leiomyoma. **A**) Serum AOPP levels and Total Fibroids Weight (g), **B**) Serum AOPP levels and infertility duration, **C**) Serum Protein Carbonyl levels and infertility duration. Nonparametric Spearman’s correlation test was used to assess correlations.

Serum AOPP levels correlated with the total fibroid weight (r=0.339; p=0.028), duration of infertility (r=0.762; p=0.006). Serum protein carbonyls also correlated with duration of infertility (r=0.683; p=0.021) (Figure 2).

## Discussion

To the best of our knowledge, this is the first report which analyses their oxidative status in sera from women with uterine leiomyoma. We found increased concentrations of serum protein carbonyl groups and AOPP in patients with uterine leiomyomas compared to control women without leiomyoma. In striking contrast, serum thiols levels were significantly lower in case of uterine leiomyoma. Our study shows a significant correlation between the levels of serum oxidative markers and clinical and anatomical features of uterine leiomyomas. Elevated serum concentrations of serum AOPP were associated with extended and heavy leiomyomas. In addition, serum AOPP and protein carbonyls correlated with the duration of infertility history.

The strength of this study lies in the novelty of the topic and in the methodological design: (i) given the high prevalence of uterine leiomyoma in the general population [3], we selected patients with well defined clinical phenotypes. Only patients with complete surgical exploration of the abdominopelvic cavity have been included in our series; (ii) all patients underwent a preoperative protocol work-up including TVUS during the month preceding the surgery. All scans were performed by a single experienced radiologist (AEM); (iii) only women with histologically proven uterine leiomyomas were allocated in the leiomyoma group; (iv) for the homogeneity of the study, the control group only included women with benign ovarian cysts, paratubal cysts or tubal defects without any evidence of uterine leiomyoma; (v) because endometriotic cells display a high endogenous oxidative stress with an increase in ROS production and alterations in ROS detoxification pathways [15,16], women with endometriosis were not included in the study; (vi) although some studies have suggested the relationship between oxidative stress and uterine leiomyoma [22–25], none of these studies have focused on the oxidative status in sera from women with leiomyoma.

In spite of all precautions, our study may be still subject to certain shortcomings and/or biases: (i) our control group consisted of women operated for benign gynaecological conditions. This may lead to biases stemming from the fact that certain of these conditions, such as tubal infertility or ovarian cysts, might be associated with altered serum oxidative stress parameters; (ii) as reactive oxygen species play important roles in the process of reproductive physiology [40,41], we assessed serum protein oxidative stress markers according to hormonal treatment and menstrual cycle phases. However 18.3% of the data on menstrual cycle phases are missing in our study. This might be worrisome because variations in estrogen balance during the menstrual cycle could influence the results of protein oxidative stress markers. This should be compensated by the homogeneity in hormonal treatment and menstrual cycle phases of the populations studied; (iii) we found positive correlations between serum AOPP and duration of infertility (r=0.762; p=0.006) and between serum protein carbonyls and duration of infertility (r=0.683; p=0.021). According to previous studies which have clearly investigated the existence of infertility in woman with uterine leiomyoma [42,43], we may speculate that the existence of higher serum oxidative protein parameters (AOPP and protein carbonyls) in case of long history of infertility may be related to ROS-mediated infertility; (iv) this study suffers from the lack of informations about the location of the uterine fibroid in the uterine wall. However, the 59 histologically proven leiomyoma patients referred to our surgery department required a surgical myomectomy because of extended uterine leiomyoma. This is highlighted by the elevated mean number of fibroids removed for each patients (6.5 ± 6.5) and the mean the mean total size (17.9 ± 11.4 cm).

Proteins are major components of serum and are one of the main targets of oxidation [44]. Oxidation alters the structure and activity of proteins. The prolonged half-life of AOPP and carbonyl groups allows an indirect reflection of the intensity of the oxidative stress. Serum protein oxidation products are elevated in several conditions in humans, including atherosclerosis, diabetes mellitus, neurodegenerative syndromes, rheumatoid arthritis or systemic sclerosis [44–46].

Examination of individual serum proteins may be useful in determining specific pathways of oxidative stress *in vivo* and the possible functional consequences of oxidative stress exposure. In this study, we determined the impact of redox imbalance on biomolecules by evaluating indirect oxidative markers as total protein carbonyl groups and AOPP and by evaluating thiols which provide an indirect reflection of the anti-oxidative defenses [44].

Total nitrates/nitrites levels are used as markers of the activity of nitric oxide synthase and the production of nitric oxide radicals [47]. We failed to show any difference in serum total nitrates/nitrites in sera of women with and without uterine leiomyoma. This result suggests that NO plays a minor role in uterine leiomyoma.

In striking contrast, we found a significant and sustained increase of serum AOPP and protein carbonyls, that are inflammatory mediators enhancing the activation of leukocytes and the oxidative stress imbalance [37,44].

Thiols contribute to the plasma antioxidant capacity and are essential for the protection from oxidative attack. In our study, the mean serum thiol level was lower in women with leiomyoma than in controls and mirrored the increase in serum carbonyls and AOPP. Thus, the accumulation of oxidized proteins is associated with the decrease of the antioxidant capacity and comforts the hypothesis of an oxidative damage in women with uterine leiomyomas.

Previous studies, based on histological analysis, have demonstrated that leiomyomas are fibrotic disorders with excessive EMC as can be observed in the skin, lung, liver and kidney [48,49]. Nevertheless, the molecular and cellular mechanisms underlying the accumulation of connective tissue remains poorly understood. Some authors consider fibrosis as a consequence of chronic inflammation [50] with sustained activation of fibroblasts and hyperproduction of collagen consecutive to the oxidative stress [51–55]. However our data do not allow to discriminate whether the observed changes in the redox status are causes or consequences of the development of uterine leiomyomas.

Given the very high prevalence and serious impact on women’s lives, uterine leiomyoma are responsible of increased healthcare costs and work loss productivity [56]. The monitoring of serum markers of the oxidative stress status could help prevent the development and growth of uterine leiomyomas.

In conclusion, our observation that serum markers of the oxidative stress correlate with the severity of uterine leiomyomas, opens new avenues in the monitoring and also the prevention of this disease.

## Supporting Information

Flowchart S1 **Longitudinal flow chart of the study population.**
(PDF)

Table S1 **Serum oxidative stress parameters in women with uterine fibroids and controls, according to previous infertility history.**
(DOCX)
