# Supplementary figures and images for: Increased Serum Oxidative Stress Markers in Women with Uterine Leiomyoma
Source: PLoS One. 2013 Aug 9;8(8):e72069. doi: 10.1371/journal.pone.0072069 (PMC3739822; doi:10.1371/journal.pone.0072069)

Flowchart showing longitudinal analysis of the study population.

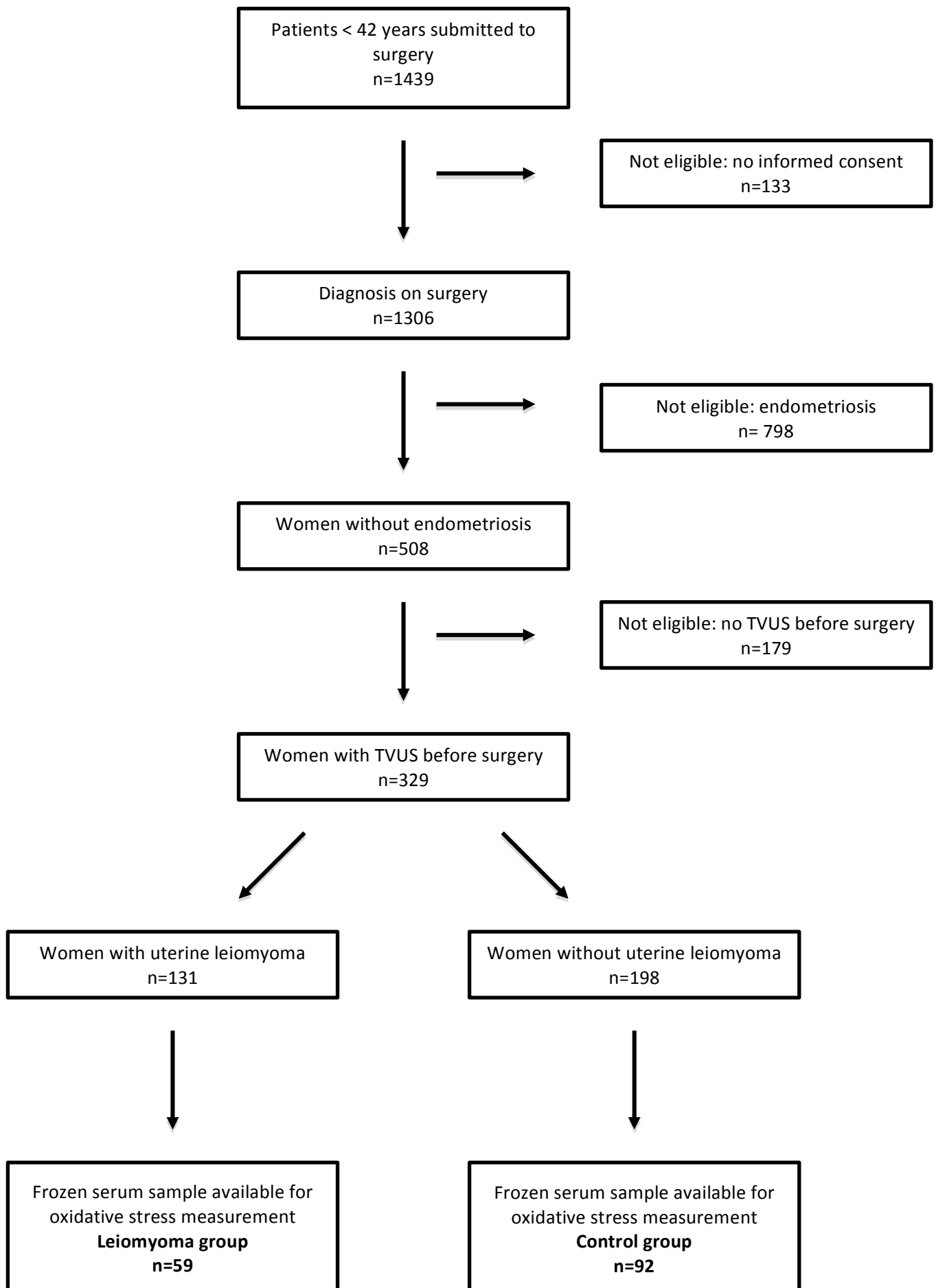

Supplement: Flowchart S1 — Longitudinal flow chart of the study population. (PDF) [file pone.0072069.s001.pdf]
