# Supplementary material for: Increased Serum Oxidative Stress Markers in Women with Uterine Leiomyoma
Source: PLoS One. 2013 Aug 9;8(8):e72069. doi: 10.1371/journal.pone.0072069 (PMC3739822; doi:10.1371/journal.pone.0072069)
Supplement: Table S1 — Serum oxidative stress parameters in women with uterine fibroids and controls, according to previous infertility history. (DOCX) [file pone.0072069.s002.docx]

| **Table S1: Serum oxidative stress parameters in women with uterine fibroids and controls, according to previous infertility history.** | | | | |
| --- | --- | --- | --- | --- |
|  |  | **Fibroids** | **Controls** | **p** |
|  |  |  |  |  |
| **Thiols (µmol/l)** | *Previous infertility history* | 360.8 (168.3 – 485.0)  n=11 | 446.3 (303.8 – 693.2)  n=36 | 0.010 ^m^ |
|  | *Without infertility history* | 409.2 (228.7 – 519.1)  n=48 | 467.0 (285.7 – 648.6)  n=56 | <0.001 ^m^ |
|  |  |  |  |  |
| **AOPP (µmol/l)** | *Previous infertility history* | 88.7 (72.8 – 290.0)  n=11 | 37.7 (10.3 – 158.5)  n=36 | <0.001 ^m^ |
|  | *Without infertility history* | 102.8 (29.8 – 261.0)  n=48 | 39.8 (18.1 – 201.2)  n=56 | <0.001 ^m^ |
|  |  |  |  |  |
| **Carbonyls (nmol/mg)** | *Previous infertility history* | 1.3 (0.4 – 2.7)  n=11 | 1.2 (0.2 – 8.6)  n=36 | 1.000 ^m^ |
|  | *Without infertility history* | 1.7 (0.0 – 3.7)  n=48 | 1.1 (0.0 – 3.4)  n=56 | 0.002 ^m^ |
|  |  |  |  |  |
| **Nitrates / nitrites (µmol/l)** | *Previous infertility history* | 16.6 (0.3 – 86.2)  n=11 | 25.7 (1.7 – 92.7)  n=36 | 0.393 ^m^ |
|  | *Without infertility history* | 22.4 (1.2 – 90.1)  n=48 | 19.7 (0.8 – 76.3)  n=56 | 0.498 ^m^ |

Data: median (range)

^m^ Statistical analyses were performed with the Mann-Whitney test

AOPP : advanced oxidation protein products
